# Aberrant Inferior Pancreaticoduodenal Artery During Upper Gastrointestinal Bleed Embolization

**DOI:** 10.7759/cureus.3945

**Published:** 2019-01-23

**Authors:** Sreeja Sanampudi, Driss Raissi

**Affiliations:** 1 Radiology, University of Kentucky College of Medicine, Lexington, USA

**Keywords:** upper gastrointestinal bleed, embolization, n-butyl cyanoacrylate, replaced right hepatic artery, aberrant inferior pancreaticoduodenal artery

## Abstract

Transcatheter arterial embolization (TAE) is a well-validated treatment for patients with non-variceal upper gastrointestinal (GI) bleeding who have failed endoscopic techniques. We present a case of a patient with duodenal ulcer bleeding that persisted despite endoscopic intervention. A gastroduodenal artery (GDA) embolization was performed; however, recurrence of bleeding warranted further embolization of inferior pancreaticoduodenal artery (IPDA). The IPDA – anterior and posterior branches – had two different origins from the middle colic artery and a replaced right hepatic artery respectively. To our knowledge, this is the first report of this IPDA branching pattern. Knowledge of common and uncommon anatomical variants of mesenteric arteries is paramount to proper interventional management of GI bleeding.

## Introduction

Upper gastrointestinal (GI) bleeding is defined as hemorrhage originating from esophagus, stomach, and duodenum proximal to Ligament of Treitz. The most common cause of non-variceal bleeding is peptic ulcer disease (PUD) [1]. Other causes include non-steroidal anti-inflammatory drugs (NSAIDs), Mallory-Weiss tears, vascular ectasias, and neoplasms [2]. First line treatment for an acute upper GI bleeding is endoscopy. Several advanced endoscopic techniques are available including mechanical therapy with clips, thermal therapy with multipolar electrocoagulation or argon plasma coagulation, and injection of various agents including vasoconstrictors and sclerosing agents. Non-endoscopic options include surgery and transcatheter arterial embolization (TAE), of which the latter is considered the gold standard for patients who fail endoscopic techniques [2, 3]. Surgery is associated with high morbidity and mortality rates, with the latter ranging from 20-40% and is a poor option for patients with multiple comorbidities and it is reserved as a last resort [4]. TAE has been shown to be a safer alternative to surgery with high percentage of technical and clinical success rates [5]. The advantages of TAE include high success rate and it eliminates the need for surgery in poor surgical candidates.

This case report brings to attention anatomical variants recognized during embolization of the inferior pancreaticoduodenal artery (IPDA). The most common anatomical branching pattern seen in the general population is the anterior and posterior IPDA sharing a common origin directly from superior mesenteric artery (SMA) [6]. However, multiple branching patterns of IPDA are present in the setting of replaced/accessory right hepatic artery (RHA) [7, 8].

## Case presentation

A 70-year-old male with history of amyotrophic lateral sclerosis (ALS), dysphagia status post gastrostomy tube placement, chronic respiratory failure status post tracheostomy, and gastroesophageal reflux disease presents with six-day duration of melanotic stools. The patient was mechanically ventilated with gastrostomy tube and tracheostomy in place. His physical exam otherwise was benign. He was found to have acute onset anemia with hemoglobin of 7.1 g/dl. He was suspected of having peptic ulcer disease secondary to NSAIDs given the history of chronic musculoskeletal pain with NSAID use. Due to continual decrease in hemoglobin requiring daily blood transfusions despite conservative management, upper endoscopy was performed. It demonstrated a bleeding ulcer distal to pylorus in the duodenal bulb. Endoscopic technique was used to remove the clot, but the procedure resulted in bleeding that could not be stopped leading to poor visualization despite epinephrine injections. Due to his multiple comorbidities, he was deemed to be a poor surgical candidate. The patient was referred to interventional radiology for embolization of gastroduodenal artery (GDA).

During the embolization of GDA, the celiac angiogram demonstrated no evidence of active extravasation, but contour irregularity at the level of the mid-GDA was noted. Prophylactic coil embolization of GDA was performed achieving GDA stasis. Post-embolization angiography of the SMA demonstrated no evidence of bleeding from IPDA branches. Incidentally noted was a replaced right hepatic artery arising from the SMA.

**Figure 1:**
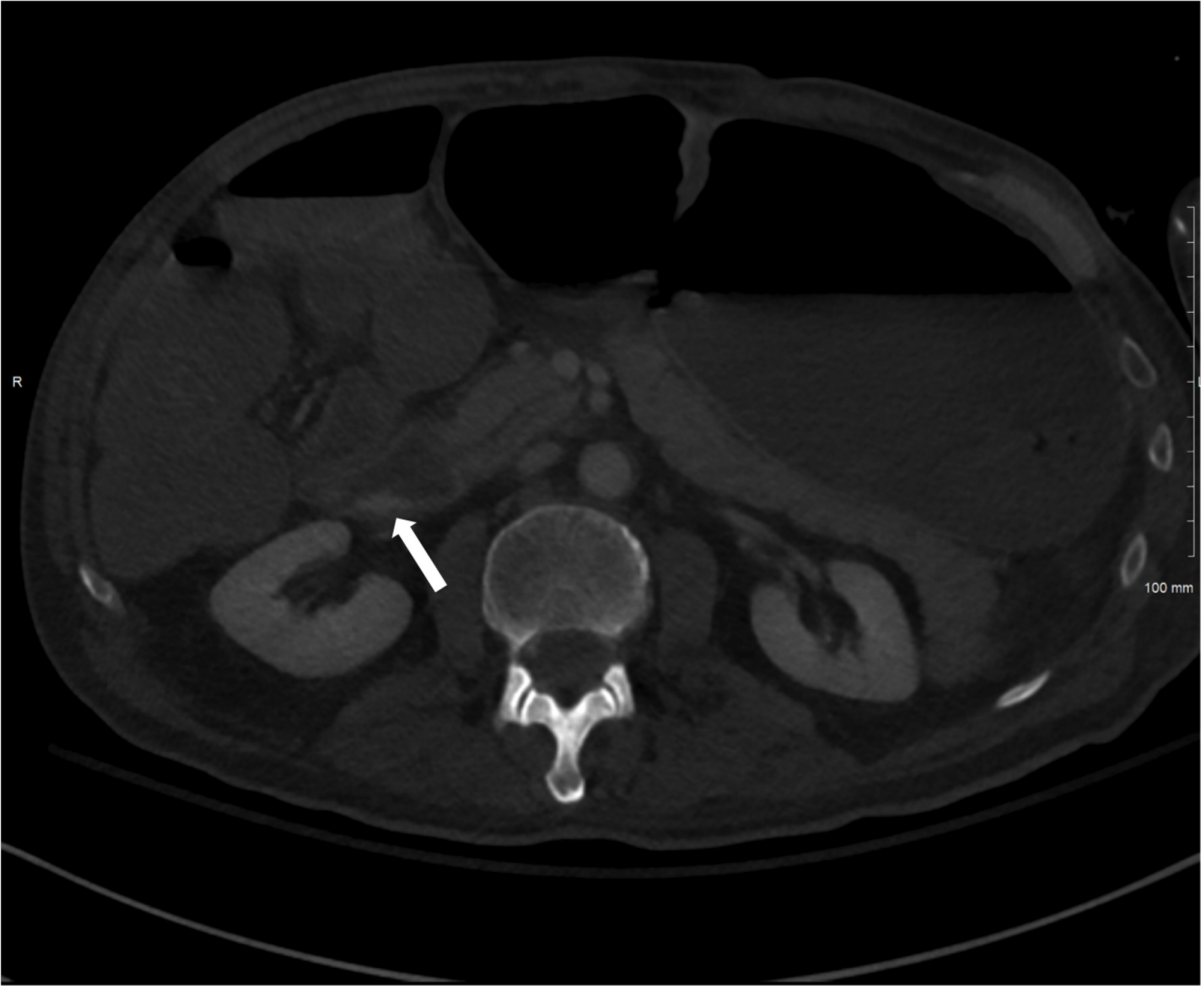
Computed tomography angiography of the abdomen. Axial contrast-enhanced abdominal computed tomography angiography demonstrates active extravasation from the posterior wall of the duodenum (white arrow).

Despite embolization of GDA, patient’s hemoglobin continued to decrease to 6.8 g/dl and the patient continued to have melena and hematemesis resulting in hypotension. The patient required an additional nine units of packed red blood cells (pRBCs), six units of platelets, and five units of fresh frozen plasma. Computed tomographic angiography (CTA) showed a large bleed from IPDA off the SMA immediately posterior to the origin of the replaced RHA (Figure 1).

The patient was referred for urgent embolization by interventional radiology.

**Figure 2:**
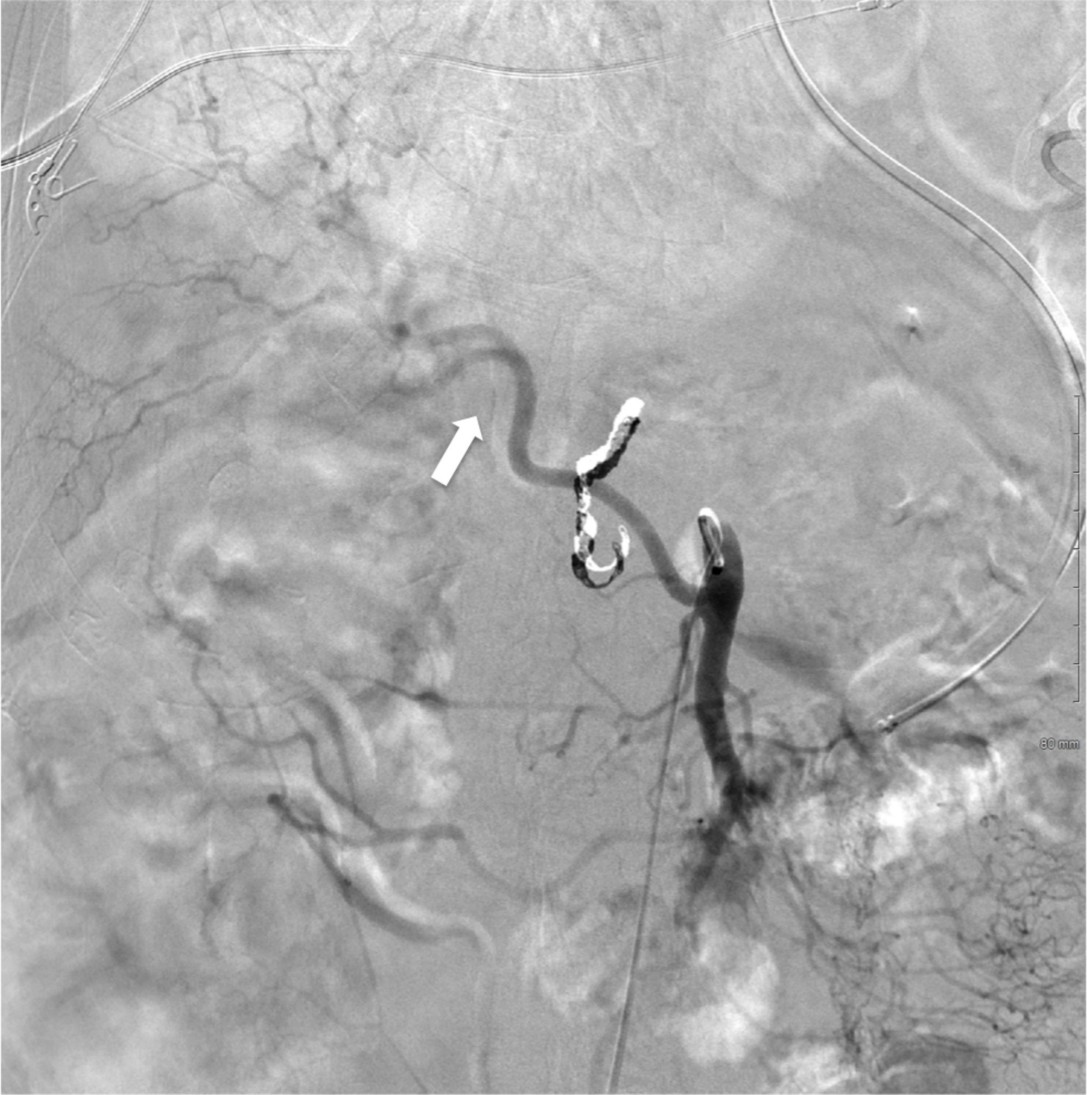
Digital subtraction angiography of superior mesenteric artery. Replaced right hepatic artery (white arrow) originates from superior mesenteric artery.

**Figure 3:**
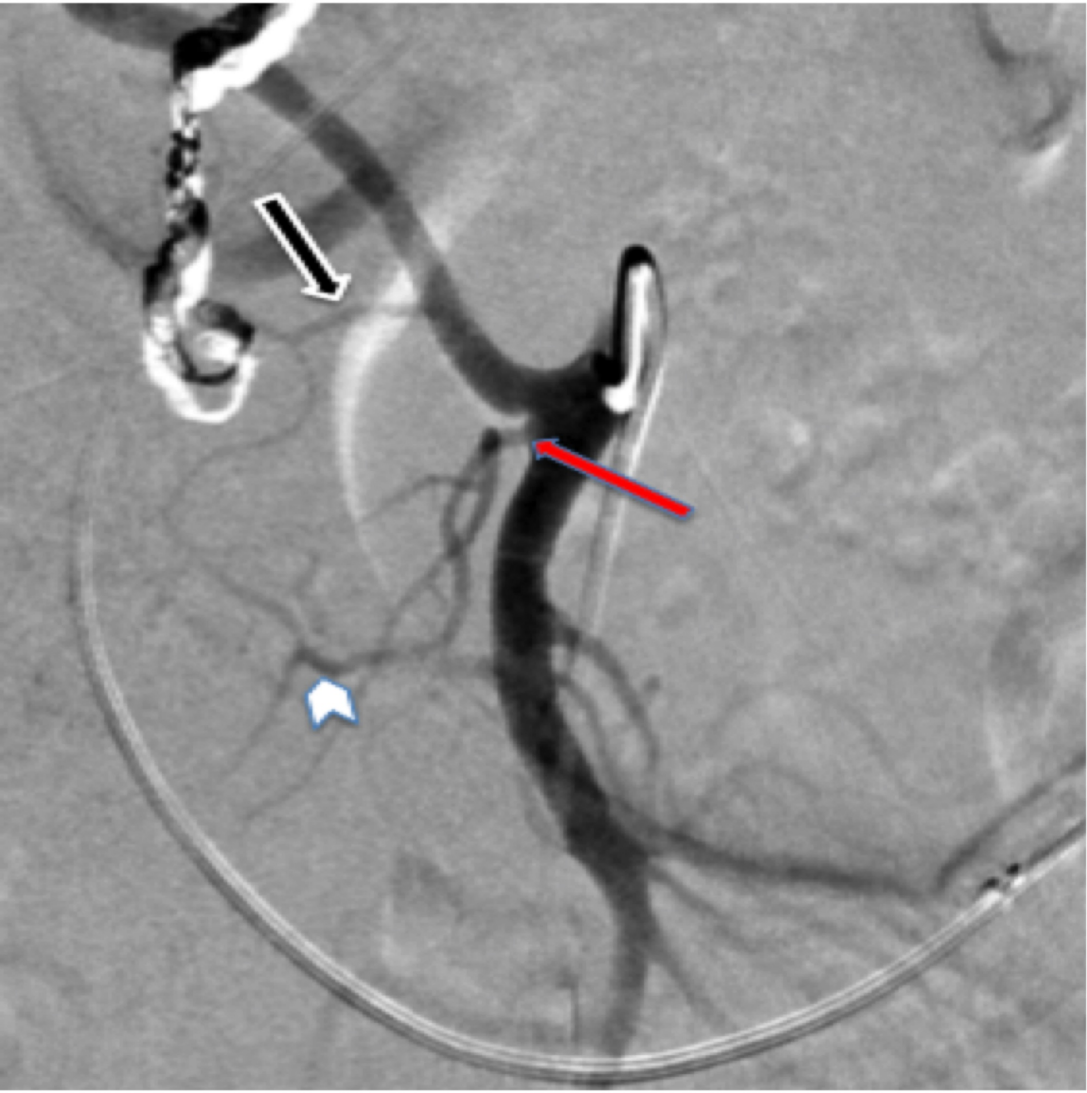
Digital subtraction angiography of superior mesenteric artery. A magnified view of Figure 2. The image demonstrates the posterior inferior pancreaticoduodenal artery (IPDA) (black arrow) originating from replaced right hepatic artery and forming an arcade with anterior IPDA arising from middle colic artery. The red arrow shows the branching point of middle colic artery and anterior IPDA (white arrow head).

During angiography, no active extravasation was identified and given that the GDA was already embolized, the most reasonable source vessel in the suspected territory would be the IPDA. The replaced RHA was selected and IPDA was identified as its first branch (Figures 2, 3).

**Figure 4:**
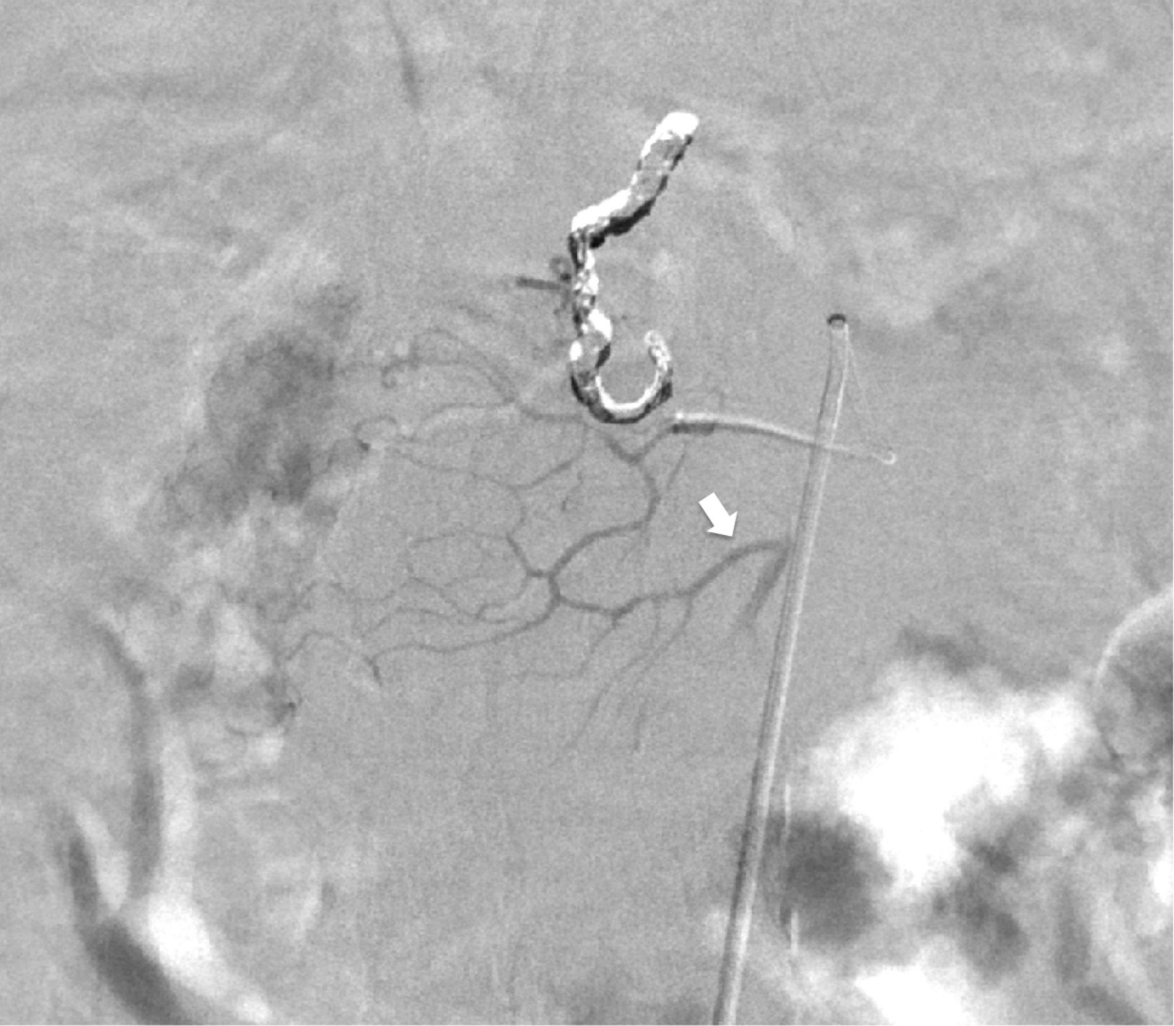
Digital subtraction angiography of the posterior inferior pancreaticoduodenal artery. Digital substation angiography of the posterior inferior pancreaticoduodenal artery (IPDA) demonstrates retrograde filling of the anterior IPDA originating from middle colic artery. White arrow depicts the branching point of anterior IPDA.

While the branch arising directly from the replaced RHA represented the posterior IPDA, the anterior IPDA was seen as collateral vessel going back to the middle colic artery (Figure 4).

**Figure 5:**
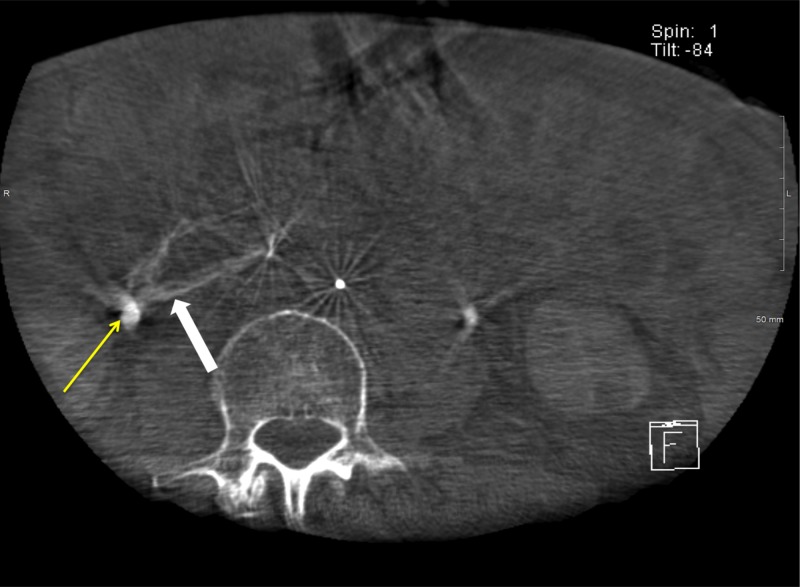
Cone beam computed tomography angiography demonstrates contrast enhancement in the duodenum. Axial cone beam computed tomography angiography of posterior inferior pancreaticoduodenal artery showing an analogous area of duodenal wall enhancement without frank extravasation (white arrow). A portion of the right ureter is also seen (yellow arrow).

However, no focus of active bleeding could be identified from digital subtraction angiography from the posterior IPDA or the anterior IPDA. Cone beam computed tomography (CBCT) catheter angiography of the posterior IPDA was performed to identify the region of the duodenal bleed analogous to the region of active extravasation seen on abdominal CTA. The same location on the posterior wall of the duodenum seen on abdominal CTA was matched on CBCT angiography (Figure 5).

**Figure 6:**
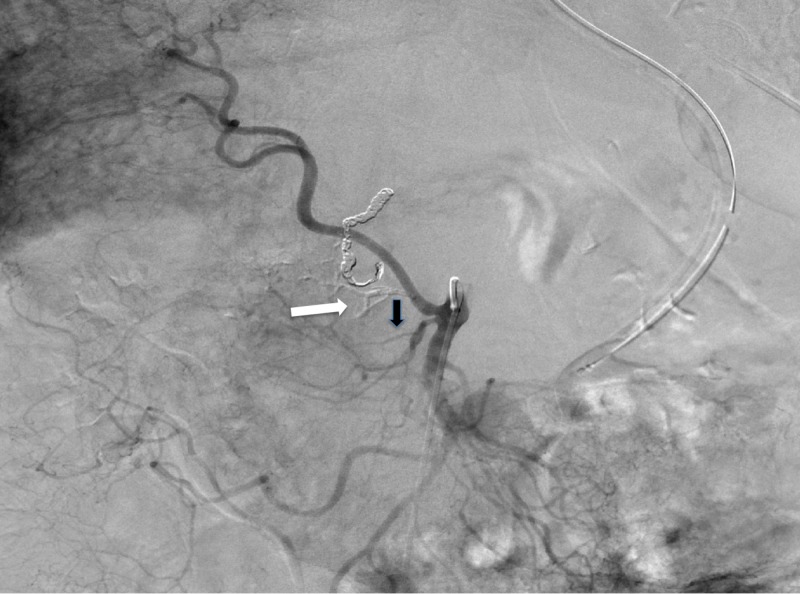
Digital subtraction angiography of superior mesenteric artery. Post-embolization angiography of the superior mesenteric artery (SMA) showing N-butyl cyanoacrylate cast formation within the embolized posterior inferior pancreaticoduodenal artery (IPDA) (white arrow). The anterior IPDA off the middle colic artery is spared (black arrow).

Subsequently, the posterior IPDA was embolized using n-butyl cyanoacrylate (NBCA) (Trufill n-BCA Liquid Embolic, Codman Neurovascular, Raynham, Massachusetts, USA) mixed with ethiodol 1:4 dilution ratio administered via the microcatheter (Figure 6).

Post-embolization angiography of the SMA demonstrated successful embolization of the posterior IPDA with sparing of the anterior IPDA. The patient became hemodynamically stable post procedure and no longer required blood products transfusion. His hemoglobin stabilized at 8.6 g/dl. The patient was successfully discharged to a skilled nursing facility to continue his recovery. At eight weeks follow-up the patient remained asymptomatic and he agreed to participate in this case report.

## Discussion

**Figure 7:**
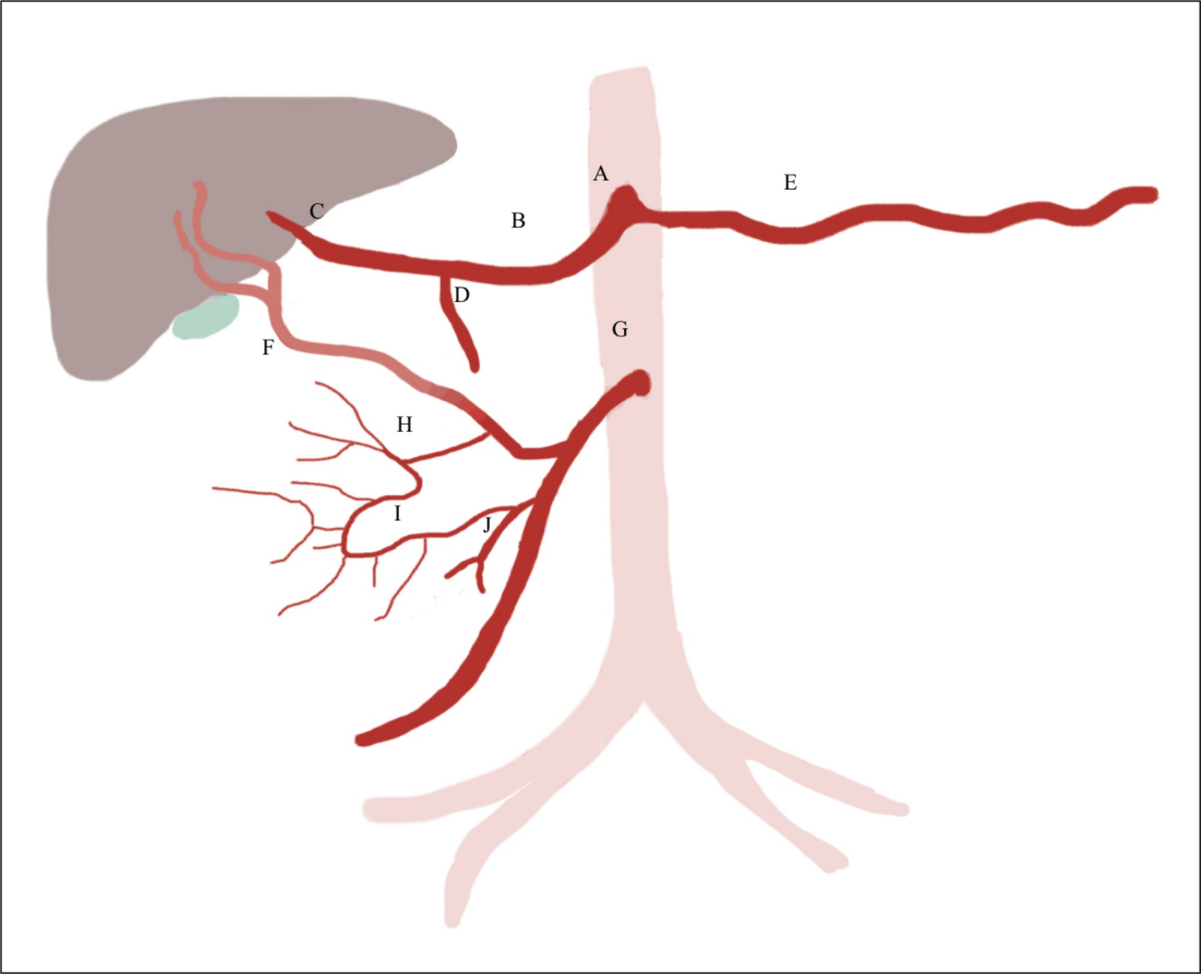
Schematic demonstrating newly described branching pattern (proposed classification D) of this aberrant inferior pancreaticoduodenal artery. Schematic demonstrating the newly described inferior pancreaticoduodenal artery (IPDA) branching variant: Anterior IPDA arising from the middle colic artery. A – celiac trunk, B – common hepatic artery, C – left hepatic artery, D – gastroduodenal artery, E – splenic artery, F – replaced right hepatic artery, G – superior mesenteric artery, H – posterior IPDA, I – anterior IPDA, J – middle colic artery.

This case sheds light on an aberrant IPDA branching pattern that has not been previously described in literature – in which the posterior IPDA originated from the replaced RHA, and anterior IPDA originates from middle colic artery. The patient had a replaced RHA originating from SMA, and the posterior IPDA was the first branch off the replaced RHA. The anterior IPDA originated from the middle colic artery, which was the second branch off the SMA. This allowed us to easily spare the anterior IPDA during embolization of the posterior IPDA. The most common anatomical pattern seen in the general population is the anterior and posterior IPDA sharing a common origin directly from SMA, which is significantly different from the aforementioned aberrant pattern [6]. The patient’s unusual anatomical variant of the IPDA is clearly depicted in Figure 7.

A replaced RHA from SMA is considered type III under Michels’ classification, and is considered the most common mesenteric vascular variant [9]. However, the presence of anterior IPDA arising from the middle colic artery and posterior IPDA branching off the replaced RHA in a single individual has not been reported in the literature to our knowledge. The shared origin of replaced RHA and IPDA has been looked at closely in the management of liver transplant patients to ensure viability of the donor and recipient organs. In one study, accessory/replaced RHA had an incidence of 12.5% with approximately one-fourth of those patients having IPDA and replaced/accessory RHA sharing a common origin from the SMA [10]. The remainder of the patients had their replaced/accessory hepatic and IPDA with two separate origins from the SMA. Other studies also reported on the branching pattern of IPDA in the setting of liver harvesting. Cherian et al. found the incidence of replaced and/or accessory RHA in 12% of their cases. Of those 12% of patients with aberrant RHA, IPDA originated from SMA in all the cases [7].

However, only one study looked at multiple branching patterns of IPDA in the setting of multiple patterns of replaced RHA. Tomimaru et al. found the incidence of replaced RHA to be 19.5% (that includes every variation of RHA – not only originating from SMA) [8]. Most commonly they found IPDA to arise directly from SMA (designated as A). The least common variation was the IPDA arising from replaced RHA (designated as C). In the third type, Tomimaru et al. describe posterior IPDA arising from replaced RHA while the anterior IPDA arises from SMA (designated as B) [8]. At this point, we would like to propose the addition of a fourth branching pattern of IPDA (can be designated as D) in which the posterior IPDA arises from replaced RHA and anterior IPDA branches off the middle colic artery instead of directly from SMA [8].

Multiple arterial anatomic variants of the IPDA have been reported in the literature, and while IPDA origin from the SMA is by far the most common pattern, interventional radiologists must be able to identify less common arterial variants. In our case, we report a previously undescribed branching pattern of the IPDA with its posterior branch arising from the replaced hepatic artery and its anterior branch arising from the middle colic artery. CBCT angiography can play an important role during angiography for problem solving, especially when challenging diagnostic and therapeutic situations arise. CBCT has been known for its role in confirming segmental distribution of hepatocellular carcinoma during chemoembolization. However, CBCT has also provided more utility in identifying GI bleeds difficult to localize with digital subtraction angiography alone [11]. A greater interval between dual phases during CBCT and higher concentrated contrast material may increase the likelihood of detecting extravasation in an active bleed. While Carrafiello et al. utilized a 15-second interval between the first and second phase of CBCT and 300 mg I/mL contrast material, Miyayama et al. utilized a 30-second interval and 370 mg I/mL contrast concentration [11,12]. While Carrafiello et al. found the active bleed in 19/20 of their patients using dual phase CBCT, Miyayama et al. found the bleed in all three of their obscure GI bleed patients [11,12].

In general, gastric and duodenal ulcers are responsible for half the cases of non-variceal upper GI bleeding [13]. Although most cases respond to endoscopic techniques such as epinephrine injection and heat probe coagulation, 5% of patients require surgery or transcatheter arterial embolization [3,14]. TAE is considered one of the first line treatments for high-risk patients in individuals with gastroduodenal bleeding after failed endoscopic techniques given the high risk of mortality and morbidity associated with surgery [14].

TAE was first introduced by Rösch et al. in the setting of non-variceal upper GI bleed. Most common indications for embolization include hemodynamic instability or massive upper GI bleed requiring at least four units of pRBCs in <24 h, failed endoscopic techniques and persistent/recurrent bleeding after surgery [15,16]. Contrast allergy and renal failure are some examples of relative contraindications that can be overcome with carbon dioxide as a contrast agent; otherwise, there are no absolute contraindications [15]. The three main types of embolization are segmental (embolization of adjacent artery(s)), localized (embolization of precise area of bleed), and proximal (embolization of parental artery) [16]. Various embolic materials have been used with the most common being metallic coils and polyvinyl alcohol. Other embolic materials include gelatin sponge and NBCA, of which the latter has been proven to be most beneficial for patients (especially those with coagulopathy) with massive bleeds and requiring expeditious embolization [16]. NBCA provides rapid and permanent embolization and has been successfully used as liquid embolic agent in embolization of GDA [17]. TAE with NBCA has been reported to be faster at achieving hemostasis than with coils or gelfoam in gastroduodenal bleeding [18].

Although several studies discussed the embolization of GDA and IPDA in treatment of an active GI bleed, few studies discussed the value of prophylactic embolization. GI bleeding is intermittent by nature and angiography often fails to demonstrate the focus of active extravasation.

To our knowledge, prophylactic embolization of IPDA has not been reported previously. In one single-center study, prophylactic embolization of GDA was employed in high-risk patients susceptible to re-bleeds after hemostasis was achieved through endoscopy [19]. Mille et al. utilized the Rockall Risk Score in addition to individual risk stratification in the determination of the patients to undergo prophylactic embolization [19]. They reported technical success rate of 98%, clinical success rate of 87%, and early re-bleeding rate of 7%, all of which support the use of prophylactic embolization in certain clinical situations. It has been reported that patients who undergo embolization have 13.3 times increased survival than patients who had a failed intervention or no intervention [20]. Most cases of re-bleeding were due to the dual blood supply to the duodenum from SMA (through pancreaticoduodenal arcade) and common hepatic artery. Most empiric embolizations were performed with metallic coils with or without gelfoam. The use of prophylactic NBCA embolization in duodenal bleeding has also been reported [19].

## Conclusions

In conclusion, we report an aberrant branching pattern of the IPDA that has not been described previously in the literature. Our report also highlights the role of prophylactic embolization of IPDA in the setting of duodenal bleeding and the added value of CBCT in such challenging situations.
